# Host factors abolish the need for polysaccharides and extracellular matrix-binding protein in *Staphylococcus epidermidis* biofilm formation

**DOI:** 10.1099/jmm.0.001287

**Published:** 2021-01-25

**Authors:** Sandra M. Skovdal, Liva Kjær Hansen, Diana Malskær Ivarsen, Guanghong Zeng, Henning Büttner, Holger Rohde, Nis Pedersen Jørgensen, Rikke L. Meyer

**Affiliations:** ^1^​ Department of Clinical Medicine, Faculty of Health, Aarhus University, Aarhus, Denmark; ^2^​ Department of Infectious Diseases, Aarhus University Hospital, Aarhus, Denmark; ^3^​ Department of Medicine, Randers Regional Hospital, Randers, Denmark; ^4^​ Interdisciplinary Nanoscience Center (iNANO), Faculty of Science and Technology, Aarhus University, Aarhus, Denmark; ^5^​ Department of Medical Microbiology, Virology and Hygiene, Universitätsklinikum Hamburg-Eppendorf, Hamburg, Germany; ^6^​ Department of Bioscience, Faculty of Science and Technology, Aarhus University, Aarhus, Denmark; ^†^​ Present address: Novo Nordisk A/S, Krogshøjvej 51, 2880 Bagsværd, Denmark

**Keywords:** *Staphylococcus epidermidis*, biofilm, aggregation, host components, extracellular matrix-binding protein (Embp), PIA

## Abstract

**Introduction:**

*
Staphylococcus epidermidis* is predominant in implant-associated infections due to its capability to form biofilms. It can deploy several strategies for biofilm development using either polysaccharide intercellular adhesin (PIA), extracellular DNA (eDNA) and/or proteins, such as the extracellular matrix-binding protein (Embp).

**Hypothesis/Gap Statement:**

We hypothesize that the dichotomic regulation of *S. epidermidis* adhesins is linked to whether it is inside a host or not, and that *in vitro* biofilm investigations in laboratory media may not reflect actual biofilms *in vivo*.

**Aim:**

We address the importance of PIA and Embp in biofilm grown in ‘humanized’ media to understand if these components play different roles in biofilm formation under conditions where bacteria can incorporate host proteins in the biofilm matrix.

**Methodology:**

*
S. epidermidis* 1585 WT (deficient in *icaADBC*), and derivative strains that either lack *embp*, express *embp* from an inducible promotor, or express *icaADBC* from a plasmid, were cultivated in standard laboratory media, or in media with human plasma or serum. The amount, structure, elasticity and antimicrobial penetration of biofilms was quantified to describe structural differences caused by the different matrix components and growth conditions. Finally, we quantified the initiation of biofilms as suspended aggregates in response to host factors to determine how quickly the cells aggregate in response to the host environment and reach a size that protects them from phagocytosis.

**Results:**

*
S. epidermidis* 1585 required polysaccharides to form biofilm in laboratory media. However, these observations were not representative of the biofilm phenotype in the presence of human plasma. If human plasma were present, polysaccharides and Embp were redundant for biofilm formation. Biofilms formed in human plasma were loosely attached and existed mostly as suspended aggregates. Aggregation occurred after 2 h of exposing cells to plasma or serum. Despite stark differences in the amount and composition of biofilms formed by polysaccharide-producing and Embp-producing strains in different media, there were no differences in vancomycin penetration or susceptibility.

**Conclusion.:**

We suggest that the assumed importance of polysaccharides for biofilm formation is an artefact from studying biofilms in laboratory media void of human matrix components. The cell–cell aggregation of *S. epidermidis* can be activated by host factors without relying on either of the major adhesins, PIA and Embp, indicating a need to revisit the basic question of how *S. epidermidis* deploys self-produced and host-derived matrix components to form antibiotic-tolerant biofilms *in vivo*.

## Introduction

The average individual carries 10–24 different *Staphylococcus epidermidis* strains [1, 2], and this common skin bacterium is the most frequently isolated pathogen from patients suffering from implant-associated infections [3, 4]. While normally a harmless skin bacterium, *S. epidermidis *’ opportunistic nature takes advantage of indwelling devices [3, 5], where it forms biofilms to become a resilient pathogen that is not susceptible to antibiotics or the immune system [6, 7].

In biofilm formation, bacteria aggregate and become encased in a protective layer of self-produced extracellular polymeric substances (EPS) [5]. Most likely, the biofilm phenotype is the natural mode of life for *S. epidermidis* under the harsh conditions on human skin [6]. Thus, *S. epidermidis *’ gene expression shifts towards the biofilm phenotype in response to environmental stimuli such as temperature, pH, salt and iron concentrations, starvation, anaerobiosis, mechanical stress and sub-inhibitory antibiotic concentrations [6, 8, 9].

The biofilm phenotype includes the production of EPS and alterations in cell metabolism. The EPS of *S. epidermidis* biofilms consist of polysaccharides, proteins, teichoic acids and extracellular DNA (eDNA), depending on the strain and environmental conditions [10]. The biofilm matrix was first thought to consist mainly of the polysaccharide intercellular adhesin (PIA), which was assumed to be crucial for biofilm formation in general [11–13]. However, it was later revealed that one-third of clinical isolates from implant-associated infections did not even contain the *icaADBC* operon required for PIA production. This led to the discovery of the large extracellular matrix-binding protein (Embp), which binds to fibronectin in the extracellular matrix of host cells and on implant surfaces [14]. *S. epidermidis* strains that do not contain the *icaADBC* operon are hypothesized to rely on Embp for biofilm formation, and this protein has been reported to control cell aggregation and biofilm accumulation [14–16]. Thus, PIA is not essential for *S. epidermidis* virulence [17].

It is estimated that more than 80 % of all clinical *S. epidermidis* isolates contains *embp* [18], which indicates a role for Embp in biofilm formation *in vivo*, possibly working in parallel with other intercellular adhesins, such as accumulation-associated protein Aap, small basic protein Sbp, serine–aspartate repeat proteins Sdr, autolysins Aae and AtlE, and elastin-binding protein Ebp [16, 17].

Several regulatory proteins have been identified to regulate the *icaADBC* loci in *S. epidermidis *, including the global regulatory proteins SarA and SigB [9]. It appears that SarA regulates the expression of Embp and PIA inversely [16], and since *sarA* is hypothesized to respond to environmental stimuli, it may enable a switch between different modes of biofilm formation, depending on the environmental conditions [10]. In standard laboratory media, SarA upregulates PIA synthesis and downregulates expression of *embp* and the proteases that activate the autolysin AtlE to cause eDNA release from lysis of neighbouring cells [16]. The environmental conditions and regulatory pathways that repress *sarA* are only partially known, but one major regulator is the alternative sigma factor B (*σ* ^B^), which is also linked to gene regulation in staphylococci in response to environmental stress stimuli [19]. *σ* ^B^ has an essential role in biofilm formation, maturation and stability, and modulates the stress response through a large regulon of genes providing a phenotype resistant to multiple stressors such as oxidative stress, antibiotics and heat [20, 21]. Thus, the bacteria have the ability to survive within the changing host milieu during infection.

Due to the inverse regulation of *embp* and *icaADBC,* it is likely that *S. epidermidis* strains carrying *icaADBC* and *embp* can switch between mutually exclusive modes of biofilm formation: a PIA-dependent biofilm mode and a PIA-independent biofilm mode involving Aap, eDNA and Embp [10]. Since Embp is a fibronectin-binding protein, we hypothesize that the dichotomic regulation of *S. epidermidis* adhesins is linked to whether it is inside a host or not. Here we investigate the significance of two major adhesins, PIA and Embp, in biofilm formation when human plasma is present, using the strain *S. epidermidis* 1585. This strain is one of many clinical isolates that contain *embp* but not the *icaADBC*, and therefore does not form biofilm in laboratory media such as tryptic soy broth (TSB) or brain heart infusion (BHI) broth unless human serum is added [14]. Using mutants that were either deficient or could be induced to express one or both of these adhesins, we studied their contribution to the aggregation and biofilm formation of *S. epidermidis* in human plasma, and the accessibility of the antibiotic vancomycin to cells in the biofilm. Our results suggest that neither PIA nor Embp are essential for biofilm formation, and that *S. epidermidis* utilizes diverse known and unknown mechanisms for biofilm formation in human plasma. Lessons learned about biofilm matrix components in laboratory media extrapolate poorly to *in vivo* conditions, and we must therefore return to the drawing board to understand even the basic concepts of how *S. epidermidis* incorporates self-produced and host-derived matrix components to form antibiotic-tolerant biofilms *in vivo*.

## Methods

### Bacterial strains

*S. epidermidis* 1585 WT and the derivative strains 1585*P_xyl/tet_embp* and 1585pTX*ica* have been described elsewhere [14–17]. The strains and their features that are relevant for this study are summarized in Table 1. The strains were cultivated on tryptic soy agar plates. The plates were supplemented with antibiotics when appropriate (as detailed in Table 1).

**Table 1.** *
S. epidermidis* 1585 strains used in this study

* S. epidermidis* strain:	Relevant biofilm-related genes	Reference
		
**1585** WT	*embp*-positive, *icaADBC*-negative, *aap*-negative clinical isolate from a port-catheter infection	[34]
**1585*P_xyl/tet_embp***	The natural *embp* promotor has been replaced with an anhydrotetracycline (200 ng ml^−1^)-inducible promotor. The resistance marker is erythromycin (200 ng ml^−1^)	[14]
**1585pTX*ica***	Contains *icaADBC* fused with xylose-inducible promoter [2 % (w/v)] on a plasmid with tetracycline (20 µg ml^−1^) as resistance marker	[14]
**1585Δ*embp***	*embp*-negative, *icaADBC*-negative	This study

### Visualization of biofilm structure

We first determined how Embp and PIA affect the biofilm structure in common laboratory media and in the presence of human plasma. Biofilms were grown statically in TSB (Sigma-Aldrich), BHI (Sigma-Aldrich) or in BHI supplemented with human plasma and studied by confocal laser scanning microscopy (CLSM). Heparin-stabilized human plasma was obtained from healthy donors at Aarhus University Hospital, pooled (from a minimum of seven donors) and stored at −80 °C until use. Bacterial cultures were streaked onto tryptic soy agar from frozen glycerol stocks and incubated 48 h at 37 °C. Single colonies were transferred to TSB or BHI in three biological replicates and incubated overnight at 37 °C, 180 r.p.m. in Erlenmeyer flasks. Cultures were diluted in fresh TSB or BHI to OD_600_=0.5 before inoculation of 90 µl culture into flat-bottom 96-well microtitre plates (μ-plate 96-well, hydrophobic untreated, IBIDI) with or without 10 or 50 % (v/v) plasma for 2 h at 37 °C. The liquid was then replaced with 90 µl fresh media of the same type, followed by 24 h incubation at 37 °C, replacement of the media, and 24 h incubation at 37 °C. Biofilms were washed gently three times with phosphate-buffered saline (PBS; Amresco) and stained with 20 µM SYTO60 (Thermo Fisher Scientific, S11342) to visualize live cells and 2 µM TOTO-1 (Thermo Fisher Scientific, T3600) to visualize dead cells and eDNA. Biofilms were visualized by confocal laser scanning microscopy (Zeiss LSM700) using a Plan-Apochromat 63×/1.40 objective, 40 µm pinholes and excitation at 639 nm for SYTO60 and 488 nm for TOTO-1. Z-stacks were captured and colour-converted so that green represents live cells and red represents dead cells and eDNA.

### Quantification of biofilms

Cultures were prepared as described above and diluted to OD_600_=0.5 in TSB, BHI or BHI+10 or 50 % (v/v) heparin-stabilized human plasma from the donor pool (healthy blood donors, Aarhus University Hospital). A peg lid (Nunc 445497 Immuno TSP lids) was inoculated in a 96-well plate (Nunc) with 160 µl culture per well for 2 h at 37 °C, 50 r.p.m. Next, the peg lid was transferred to a new 96-well plate containing fresh media of the same type, incubated 24 h at 37 °C and 50 r.p.m., transferred to a new 96-well plate with fresh media, and incubated for another 24 h at 37 °C and 50 r.p.m. Biofilms on the pegs were then quantified by safranin staining as described elsewhere [22]. In brief, the peg lid was air-dried at room temperature for 30 min, stained for 10 min in Gram’s safranin solution (Sigma-Aldrich), rinsed in demineralized water and air-dried for 30 min. The safranin stain was then extracted into 33 % (v/v) acetic acid for 30 min at room temperature, and the absorbance was measured at 530 nm (Holm and Halby, BioTex, Power Wave XS2). A D’Agostino–Pearson omnibus test was used to determine normality (alpha=0.05), and a Welch’s unequal variance *t*-test was used to determine statistical significance. The differences were considered statistically significant if *P*<0.05.

### Mechanical properties of *S. epidermidis* biofilms expressing PIA and Embp

To investigate how PIA and Embp affect the mechanical properties of the biofilm, the Young’s modulus (elasticity) of biofilms was calculated using atomic force microscopy (AFM) nanoindentation measurements. *S. epidermidis* 1585 strains were prepared as described above, and biofilms were grown on glass slides (Superfrost Ultra Plus, Thermo Fisher Scientific) submerged in growth media for 24 h at 37 °C. Biofilms were briefly dried in air and rehydrated by adding PBS. AFM nanoindentation was performed with 60 µm glass microbeads glued on tipless cantilevers (spring constant 0.03 N m^−1^, HQ:CSC38/TIPLESS/NO AL, from MikroMasch Europe), using a loading force of 5 nN, a Z length of 2–3 µm and a loading speed of 1 µm s^−1^. The preparation of probes, calibration of AFM and calculation of Young’s modulus were performed according to a published procedure [23]. Ten measurements were performed on each of the five random locations on each of three replicate biofilm samples. Statistical calculations were performed using GraphPad Prism 7.0 (GraphPad Software, Inc., USA). A Welch’s unequal variance *t*-test was used to determine statistical significance. The differences were considered statistically significant if *P*<0.05.

### Quantification of aggregate formation in response to human plasma or serum

Biofilms formed in human plasma were only loosely attached to the surface, and we therefore wanted to quantify the time-dependent formation of suspended aggregates in response to exposure to host factors. The size distribution of suspended aggregates was determined by bright-field microscopy (Carl Zeiss Axio Vert.A1) and image analysis (ImageJ version 1.51 [24]) after adding human serum or plasma to *S. epidermidis* 1585 WT cultures. *S. epidermidis* cultures were prepared as described above and concentrated to OD_600_=5, and 20 µl was used to inoculate a microtitre plate containing 180 µl BHI, or BHI with 10 or 50 % (v/v) human plasma, 10 % (v/v) human serum (from healthy donor pool, Aarhus University Hospital) to reach a final OD_600_=0.5. To identify a possible role for specific human matrix proteins, aggregation was quantified in BHI with physiological concentrations of magnesium and calcium amended with 10 % (v/v) human plasma that was pretreated with 200 U ml^−1^ collagenase (Sigma-Aldrich), 500 U ml^−1^ streptokinase (Sigma-Aldrich), or 20 U ml^−1^ elastase (Sigma-Aldrich) for 4 h at 37 °C, 100 r.p.m. Aggregation was quantified after 24 h incubation at 24 h at 37 °C, 50 r.p.m. For the time lapse experiment, aggregation was quantified after 0, 5, 15, 30, 60, 120 min and 24 h at 37 °C, 50 r.p.m. For each biological replicate, at least 20 images were captured at random locations with a 20× objective. A Mann–Whitney test was used to determine statistical significance between the mean aggregate size for each sample (*P*<0.05).

### Vancomycin susceptibility

To address whether PIA and Embp in the biofilm matrix affected antibiotic tolerance we determined the minimal biofilm eradication concentration (MBEC) for biofilms grown on the bottom of 96-well plates and of aggregates in the suspension. Overnight cultures were prepared for the inoculation of biofilms as described above, diluted in fresh BHI to OD_600_=1 and diluted 10-fold upon inoculation into flat-bottom 96-well microtitre plates (Nunclon Delta Surface, Thermo Fisher Scientific) containing BHI with or without 10 % (v/v) human plasma, before being incubated for 24 h at 37 °C, 50 r.p.m. Then, serial dilutions of vancomycin were added the wells to reach final concentrations of 2048, 1024, 512, 256, 128, 64, 32, 16, 8 and 4 mg l^−1^, and the plate was incubated for 24 h at 37 °C, 50 r.p.m. The supernatant was then transferred to Eppendorf tubes, centrifuged for 3 min at 2000 ***g*** and replaced by fresh BHI, and then incubated for 24 h at 37 °C, 50 r.p.m. The biofilms remaining in wells were amended with fresh BHI and incubated for 24 h at 37 °C, 50 r.p.m. If any cells in the biofilm or in suspended aggregates had survived vancomycin exposure, planktonic cultures would grow during the incubation. Subsequently, 10 µl from each sample was plated onto BHI agar and incubated 24 h at 37 °C to determine bacterial survival.

### Vancomycin penetration in biofilms

Matrix components may affect biofilm survival by preventing penetration of antibiotics. We therefore investigated vancomycin penetration in biofilms expressing PIA or Embp. Biofilms were cultivated in 96-well plates (μ-plate 96-well, IBIDI) for 48 h as described above and stained for 30 min by replacing the supernatant with PBS containing 3 μg ml^−1^ BodipyFL vancomycin (Thermo Fisher Scientific), 10 µM SYTO41 (Thermo Fischer Scientific) and 6 µM propidium iodide (Thermo Fischer Scientific), followed by CLSM imaging with a Plan-Apochromat 63×/1.40 oil objective and excitation at 405, 488 and 555 nm.

## Results

*S. epidermidis* 1585 is known to be biofilm-negative in laboratory media, but to form biofilm in the presence of human serum. In this study, we sought to determine the significance of two major matrix components of *S. epidermidis* biofilms – PIA and Embp – in the presence of human plasma, and to pinpoint whether specific human extracellular matrix proteins are required for aggregation and biofilm formation.

### Biofilms form as suspended aggregates in the presence of human plasma

**Fig. 1.**
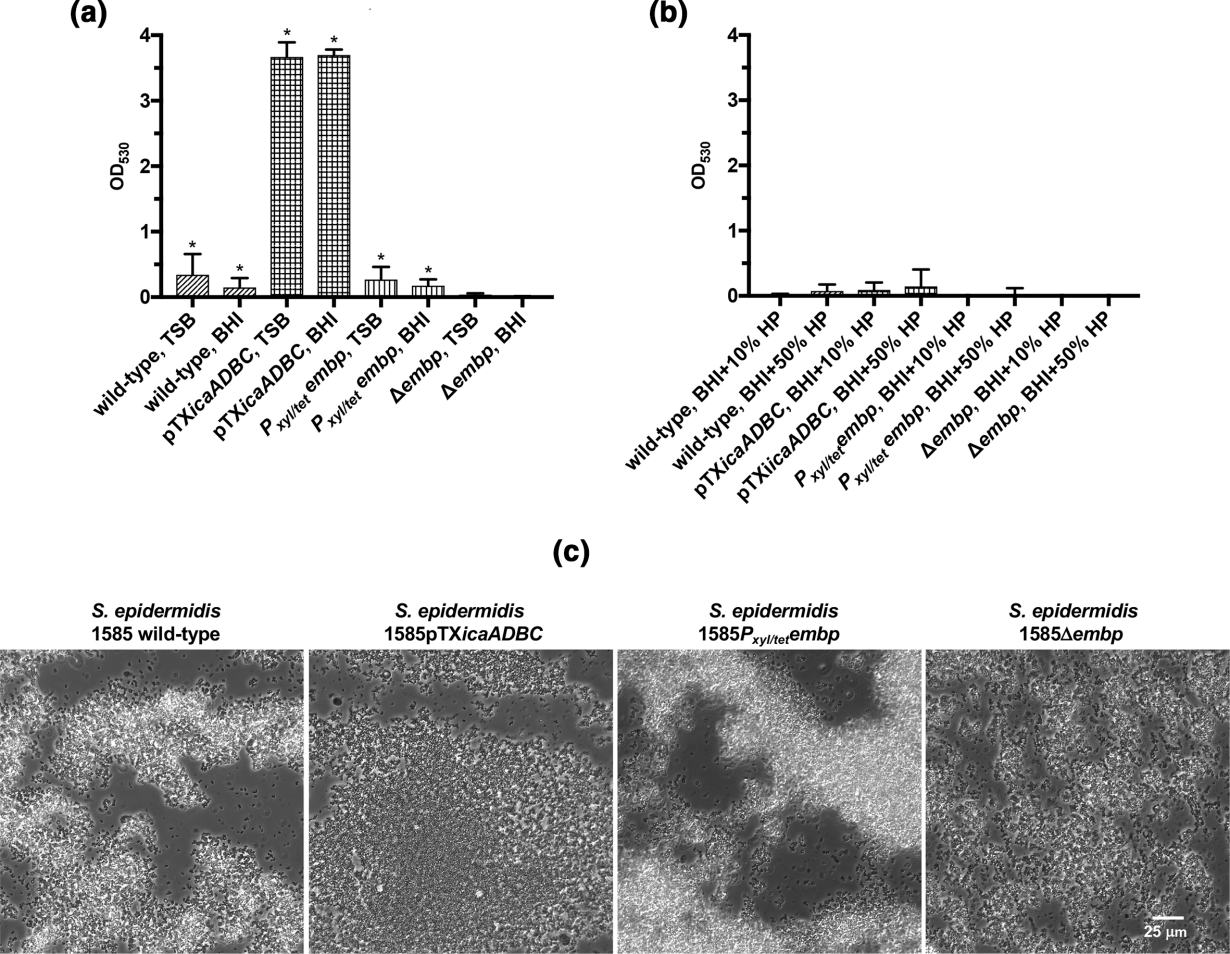
Quantification of biofilms on peg lids by safranin staining. Biofilm formation on peg lids quantified by safranin staining of the wild-type *S. epidermidis* 1585, or strains with induced PIA production (* S. epidermidis* 1585pTX*icaADBC*), induced Embp production (* S. epidermidis* 1585*P_xyl/tet_embp*), or deficiency of both PIA and Embp production (* S. epidermidis* 1585Δ*embp*). Strains were grown in (a) laboratory media (TSB or BHI) or (b) BHI containing 10 or 50 % (v/v) human plasma (HP) (*P<0.05, Welch’s *t*-test). (**c**) Phase-contrast microscopy of suspended aggregates formed by the *S. epidermidis* 1585 strains in BHI containing 50 % (v/v) human plasma.

Quantification of biofilms on peg lids, using the standard 96-well assay, showed that biofilm formation only occurred in the absence of human plasma, and when PIA production was induced (Fig. 1a, b). However, the lack of biofilm formation is in contrast to previous findings that demonstrated biofilm formation in serum [14]. We therefore hypothesized that biofilms had formed as suspended aggregates, which were not quantified in the assay due to poor adherence to the polystyrene surface. Indeed, bright-field microscopy revealed large suspended aggregates in the presence of human plasma, regardless of the presence or absence of PIA or Embp (Fig. 1c). Hence, the results from standard *in vitro* biofilm assays can be highly biased if they only quantify attached biofilms.

**Fig. 2.**
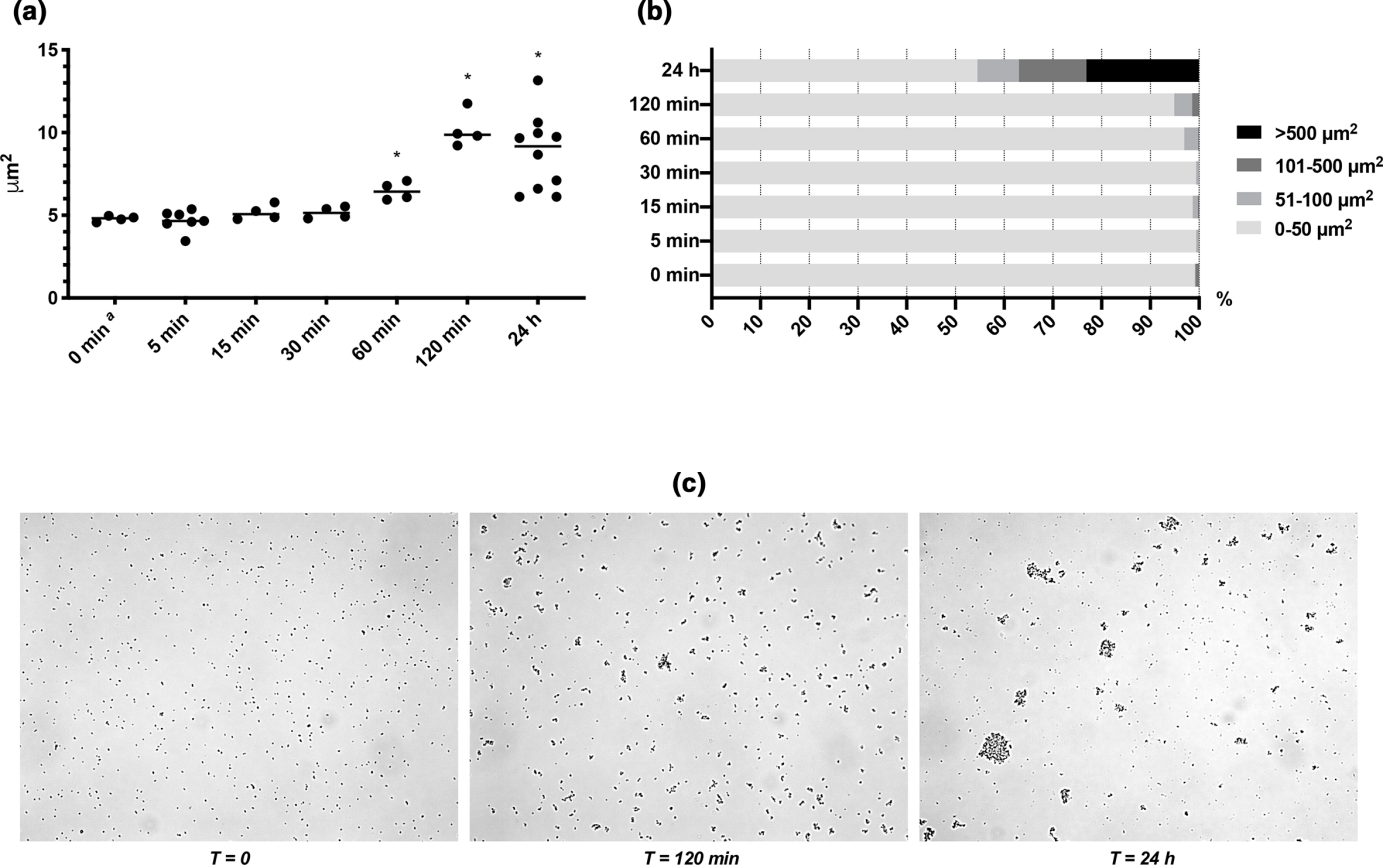
Formation of aggregates after addition of human plasma. (a) The average size of aggregates formed by *S. epidermidis* 1585 WT increases significantly at 1 h after the addition of 10 % (v/v) human plasma [*P<0.05 (two-tailed), Mann–Whitney *U*=0; median]. One data point represents one biological replicate and 20 technical replicates with >20000 measurements. (**b**) Aggregate size distribution in time after the addition of human plasma (% of biomass) reveals the emergence of large aggregates after 24 h, although this is not statistically significant due to the large variation [Mann–Whitney test (two-tailed), *P*>0.05]. (**c**) Phase-contrast microscopy of suspended aggregates formed over time by *S. epidermidis* 1585 in BHI with 10 % (v/v) human plasma.

Aggregates form through interactions that connect cells via surface-bound adhesins, or simply by bridging or depletion aggregation mediated by electrostatic interactions between the cells and charged polymeric molecules in the surrounding liquid [25]. We hypothesized that the type of aggregation is indicated by how fast aggregation occurs, since colloidal aggregation can be immediate, while receptor–ligand interactions may require synthesis and secretion of the necessary adhesins. Time-dependent analysis of aggregate formation showed that aggregates appeared after 2 h incubation in 10 % (v/v) plasma, and the aggregates grew in size and became more abundant after 24 h (Fig. 2). We repeated the experiment in 100 % (v/v) plasma, in case the concentration of glycosylated proteins was too low to cause depletion aggregation in 10 % (v/v) plasma. However, results were similar in the two experiments (Fig. S1, available in the online version of this article). The timing of aggregate formation indicates that *S. epidermidis* responds to the presence of host factors and changes phenotype, rather than aggregating through e.g. depletion aggregation.

**Fig. 3.**
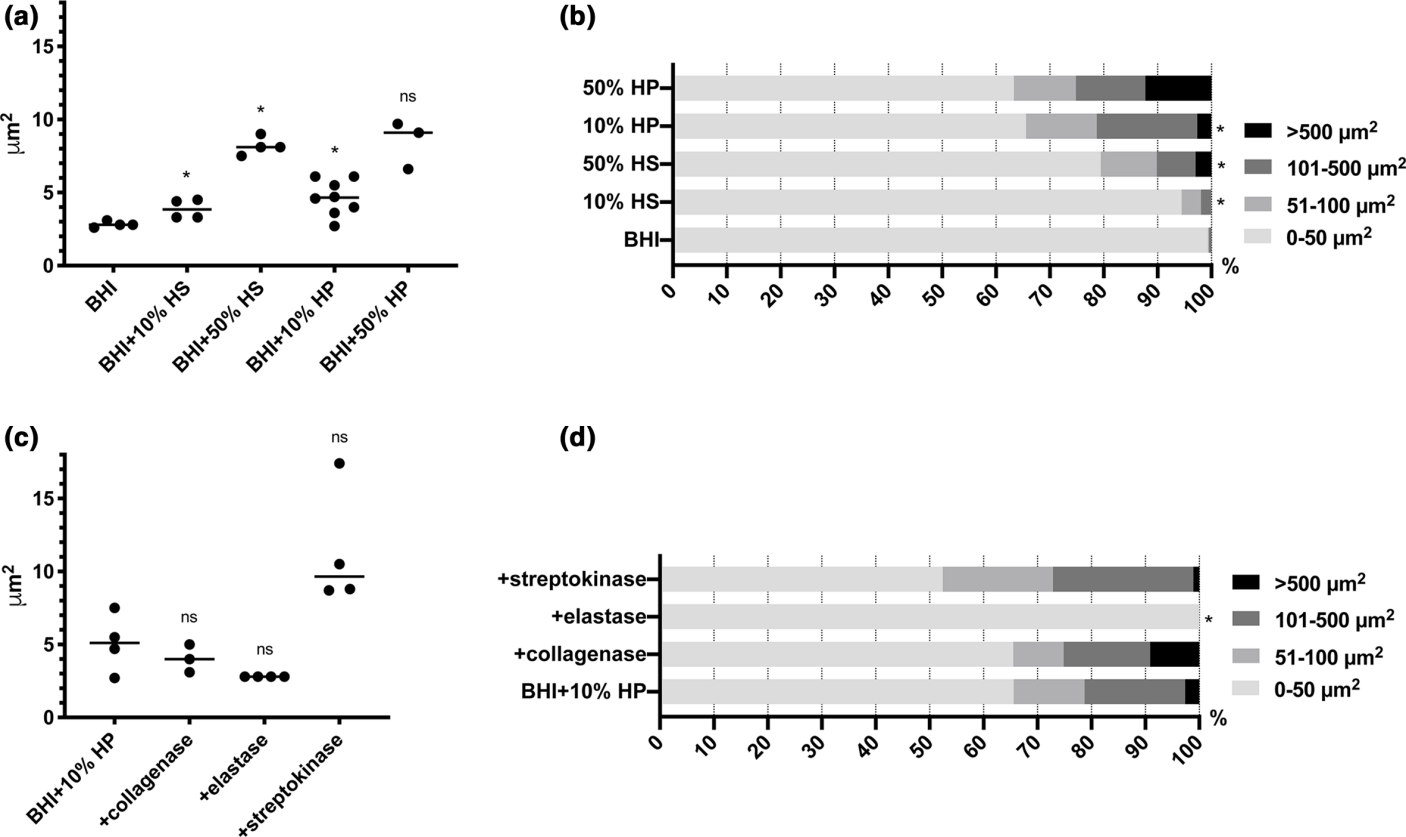
Aggregation of *S. epidermidis* 1585 after the addition of serum, plasma, or enzyme-treated plasma. (a) The average aggregate size by *S. epidermidis* 1585 WT increased significantly 24 h after amending BHI 10 or 50 % (v/v) human serum (HS), or 10 or 50 % (v/v) human plasma (HP) [*P<0.01 (two-tailed), Mann–Whitney *U*=0; median; ns, not significant]. (**b**) Aggregate size distribution analysis revealed the emergence of large aggregates 24 h after the addition of human plasma (% of biomass) [*P<0.05 (two-tailed), Mann–Whitney *U*=0; median]. (**c**) Pretreatment of human plasma with collagenase, elastase, or streptokinase for 4 h did not diminish the effect of plasma on average aggregation [*P*=0.17, 0.71 and 0.38 (two-tailed), Mann–Whitney *U*=3, 7 and 5; median; ns, not significant]. (**d**) However, aggregate size distribution analysis revealed that elastase prevents the emergence of large aggregates >50 µm^2^ [*P<0.05 (two-tailed), Mann–Whitney *U*=0; median]. One data point represents one biological replicate and 20 technical replicates with >20000 measurements.

*S. epidermidis* is equipped with an array of surface-bound adhesins that interact specifically with human extracellular matrix proteins present in human plasma, such as fibrinogen (SdrG), fibronectin (Embp), vitronectin (Sdr), elastin (Ebp) and collagen (SdrF) [3]. We speculated that some of these proteins may act as intercellular adhesins and thereby promote aggregation. For example, the glycosylated protein fibrinogen is highly abundant in plasma and is involved in cell aggregation in other contexts [26, 27]. Serum does not contain fibrinogen, and we therefore compared aggregate formation in serum and plasma to investigate a potential role of fibrinogen. Both plasma and serum induced aggregation (Fig. 3a), but aggregates were larger and more abundant in the presence of plasma (Fig. 3b), indicating that fibrinogen plays a role. We therefore investigated whether the addition of fibrinogen to serum or BHI could induce aggregation. However, this was not the case (Fig. S2), and if fibrinogen does promote aggregation of *S. epidermidis* in plasma, it is only one of multiple factors.

Many of the human extracellular matrix proteins can fibrillate, and we therefore tested whether fibrillar forms of these proteins assisted bacterial aggregation. Interestingly, enzymatic degradation of protein fibrils by pretreatment of human plasma with elastase prevented bacterial aggregation, but this was not the case for collagenase (targeting collagen) or streptokinase (targeting fibrin) (Fig. 3c, d). Elastase degrades elastin, but its proteolytic activity is not very specific, and it may have acted on human plasma proteins as well as bacterial cell wall-anchored proteins used in aggregation.

### Neither PIA nor Embp are required for biofilm formation in human plasma

We could not pinpoint a single specific human matrix protein as the critical component for biofilm formation in plasma, and then turned our attention to the major adhesins produced by the bacteria. As mentioned above, the production of PIA and Embp is inversely regulated through SarA, leading to *embp* upregulation and *icaADBC* downregulation in human serum [14]. This suggests that the fibronectin-binding Embp is important for biofilm formation when *S. epidermidis* is in the bloodstream or tissue, while PIA is used in other environments. We therefore investigated whether either PIA or Embp were a prerequisite for biofilm formation when human plasma is present, and how PIA or Embp affected the properties of the biofilm matrix.

**Fig. 4.**
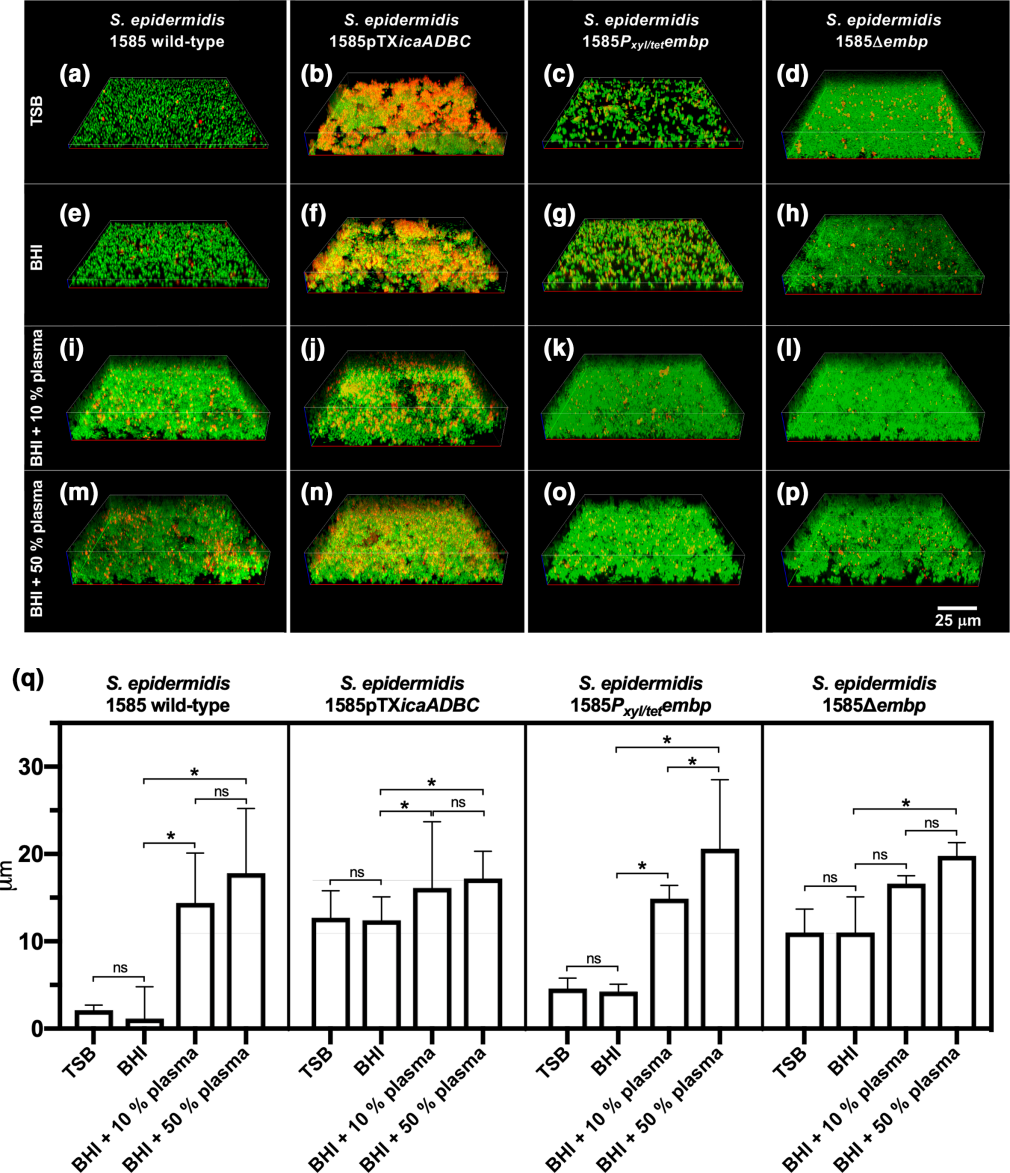
Biofilm structure and thickness. 3D CLSM images of *S. epidermidis* 1585 WT and mutant strains grown in TSB, BHI, BHI with 10 % (v/v) plasma, or BHI with 50 % (v/v) human plasma (**a–p**). Images show bacteria (green) and extracellular DNA (red). Evaluated by visualization and quantification of biofilm thickness (**q**), *S. epidermidis* 1585 WT and 1585*P_xyl/tet_embp* did not form biofilm in TSB and BHI, unless amended with 10 or 50 % (v/v) human plasma [*P*<0.05 (two-tailed), Mann–Whitney test]. *S. epidermidis* 1585pTX*ica* and 1585Δ*embp* formed biofilm in all four media, but made significantly thicker biofilms when human plasma was added [*P*<0.005 (two-tailed), Mann-Whitney test]. The bars represent the median and range of at least three biological replicates with at least three technical replicates.

We visualized biofilms formed by *S. epidermidis* strains that either lacked PIA and/or Embp, or expressed it from an inducible promotor, thereby decoupling the effect of plasma on gene expression. Biofilms were grown in laboratory media or in the presence of plasma to address whether the role of these major bacterial adhesins was different when human matrix proteins were available (Fig. 4). As expected, the PIA-deficient *S. epidermidis* 1585 WT did not form biofilm in laboratory media without plasma (Fig. 4a, e, i, m). Induction of PIA production from a plasmid strongly promoted biofilm formation in laboratory media, resulting in a biofilm matrix that is rich in eDNA (Figs 4b, f, j, n and S3), which presumably co-localizes with PIA as previously shown [28].

Although PIA production had a dramatic impact on biofilm formation in laboratory media, the effect was insignificant in plasma (Fig. 4), suggesting that polysaccharides may be redundant for biofilm formation *in vivo*. Induction or deletion of *embp* also made no apparent difference to the biofilm structure and thickness in the presence of plasma (Fig. 4k, l, o, p). This fibronectin-binding adhesin may play a role in microbe–host interactions, but our results show no indication that Embp is required for the formation of a biofilm. Interestingly, the deletion of *embp* stimulated biofilm formation in TSB and BHI (Fig. 4d, h), although the amount of biofilm formed was not sufficiently adherent to polystyrene to allow detection in the peg lid biofilm assay (Fig. 1a). Ultimately, we concluded that neither PIA nor Embp are essential for *S. epidermidis* biofilms in human plasma.

### Physical properties and antibiotic penetration in biofilms

**Fig. 5.**
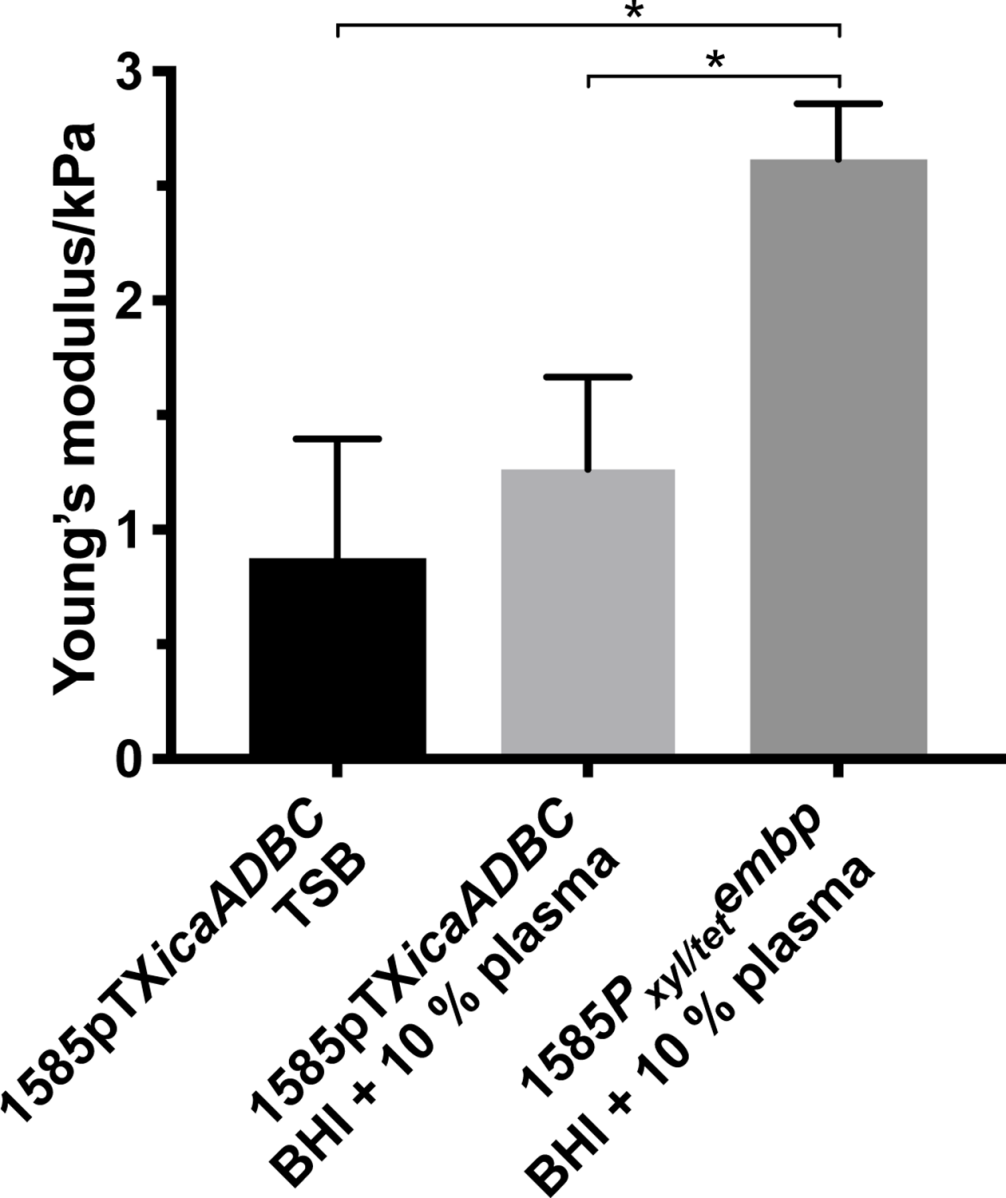
Mechanical properties. Young’s modulus (elasticity) in kPa and standard deviation (sd) of *S. epidermidis* 1585 biofilms, calculated from AFM nanoindentation. Biofilms in which PIA production was induced [* S. epidermidis* 1585pTX*icaADBC* grown in TSB and *S. epidermidis* 1585pTX*icaADBC* grown in BHI with 10 % (v/v) human plasma (HP)] were less stiff than biofilms in which only Embp was induced [(* S. epidermidis* 1585*P_xyl/tet_embp* grown in BHI with 10 % (v/v) plasma] [Welch’s *t*-test, ±sd, *t*(df)]: 0.876±0.521, *n*=3, *P*<0.05; and 1.263±0.403, *n*=3, *P*<0.05; compared with 2.616±0.243, *n*=3. Each sample (*n*) represents 1 biological replica with 5 measurement points and 10 force curves at each point.

Although the biofilms appeared to be visually similar, we hypothesized that expression of PIA or Embp would lead to very different matrix structures and therefore different physical properties. To quantify this, we measured the biofilms’ mechanical properties by AFM nanoindentation. Biofilms with PIA expression had the highest elasticity (i.e. the lowest Young’s modulus), while the same strain with naturally induced Embp (in BHI+human plasma medium) had lower elasticity, and biofilm without PIA expression and with artificially induced Embp expression had the lowest elasticity (Fig. 5). These differences confirm that polysaccharide-rich biofilms are more elastic than biofilms with a primarily proteinaceous matrix. This is consistent with the fact that polysaccharides are more elastic than proteins due to the loose high-order confirmations defined by hydrophilic molecule chains.

**Fig. 6.**
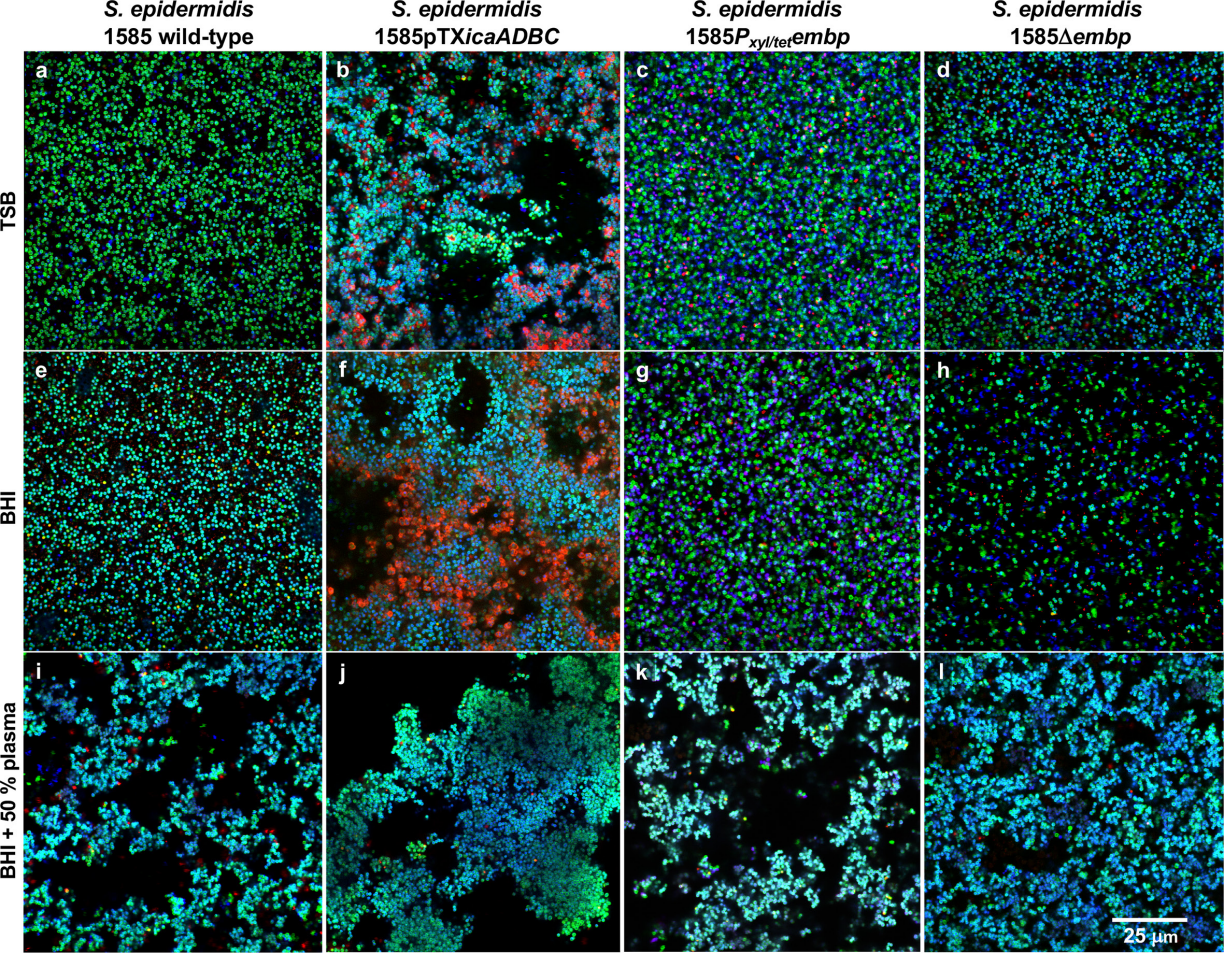
Vancomycin penetration of biofilms depending on PIA or Embp CLSM showing vancomycin binding in the bottom layer of the biofilms formed by *S. epidermidis* 1585, 1585*P_xyl/tet_embp*, 1585pTX*icaADBC* and 1585Δ*embp* in TSB, BHI, BHI with 50 % (v/v) human plasma. Blue, intracellular DNA; red, extracellular DNA; green, BodipyFL vancomycin.

The different structural and physical properties of biofilms that produce PIA and/or Embp in the presence or absence of host factors raised the question of how these differences affected the protective properties of the biofilm matrix against antibiotics. Limited antibiotic penetration is proposed as one of the causes of biofilms’ antibiotic tolerance to e.g. vancomycin [29, 30]. We visualized vancomycin penetration and binding to the cell wall of bacteria in biofilms of the four strains grown in the absence or presence of human plasma. Fig. 6 shows 2D images of the bottom layer of cells in the biofilms. We observed cell-to-cell variation in vancomycin binding, but there was no indication of limited vancomycin penetration in the biofilm (Fig. 6). Nevertheless, the antibiotic tolerance was far beyond physiological levels for all biofilms (Table 2). Taken together, these results suggest that the antibiotic tolerance cannot be ascribed to a shielding function of the matrix, but rather the presence of persister cells that tolerate high antibiotic levels.

**Table 2.** Minimal biofilm eradication concentration (MBEC) for vancomycin against biofilms or suspended aggregates of *S. epidermidis* expressing PIA or Embp. Biofilms were formed by induction of PIA production in *S. epidermidis* 1585pTX*icaADBC* or Embp production in *S. epidermidis* 1585*P_xyl/tet_embp* in either BHI or BHI with 10 % (v/v) human plasma (BHI+HP).

* S. epidermidis* 1585 strain:	Aggregates (BHI)	Aggregates (BHI+HP)	Biofilm in wells (BHI)	Biofilm in wells (BHI+HP)
**pTX*icaADBC***	>2048 mg l^−1^	>2048 mg l^−1^	>2048 mg l^−1^	>2048 mg l^−1^
***P_xyl/tet_ embp***	>2048 mg l^−1^	>2048 mg l^−1^	>2048 mg l^−1^	>2048 mg l^−1^

## Discussion

Much knowledge about *S. epidermidis* biofilms has been obtained from *in vitro* studies. Here, we show that host components in human plasma cause fundamental changes in biofilm formation. *In vitro* models used for understanding biofilm infections must therefore, as a minimum, include host factors in the growth media. We show that some of the matrix components previously deemed essential for biofilm formation turn out to be redundant when host factors are present. The central role previously ascribed to PIA appears to be an artefact from growing the biofilms in sugar-rich laboratory media. Indeed, production of PIA is downregulated in human serum [14], and the *icaADBC* operon responsible for its production is absent in a substantial proportion of clinical *S. epidermidis* isolates [14]. In contrast to PIA, Embp is present in almost all clinical isolates, here among *S. epidermidis* 1585, and it is upregulated when *S. epidermidis* is grown in human serum [14], suggesting that Embp plays a role in the pathology of *S. epidermidis* biofilm infections. This may be the case, but our study shows that it is not a prerequisite for biofilm formation per se. It does, however, affect biofilm elasticity, and its fibronectin-binding properties suggests a role in attachment to host tissue. The detailed role of Embp in the biofilm matrix remains elusive.

Previous research on transcriptional regulation of PIA and Embp suggested that *S. epidermidis* shifts between an Embp/eDNA/Aap-mediated biofilm mode in the presence of human serum, and a PIA-mediated biofilm mode in the absence of human serum [16]. Although we did not study gene regulation, confocal microscopy revealed copious amounts of eDNA in the biofilm matrix if PIA production was induced, and human plasma was absent (Figs 4b, f, j, n and S3). This is in contrast to the previous findings by Christner *et al*. [16] and suggests that eDNA is released under a variety of conditions. Furthermore, we conclude that the accumulation of eDNA in the biofilm matrix depends not only on autolysin-induced release, but also on the availability of matrix components (e.g. PIA) that retain the released DNA.

We observed that biofilms grown with human plasma adhered poorly to the abiotic substrate. This highlights an important technical issue, namely that suspended biofilms are not easily quantified by standard laboratory assays. High-throughput screening assays only quantify attached biofilms and will therefore deem the observed strains biofilm-negative, although time-consuming low-throughput microscopy of biofilms prepared more gently reveals such biofilms. Used as diagnostic tools, such assays could result in misinterpretation and underestimation of the infection and the pathogen itself.

*S. epidermidis* produces many different adhesins that bind to host proteins, and it is generally assumed that these adhesins facilitate attachment to implanted medical devices by interacting with adsorbed host proteins on the implant surface. The lack of adhesion in the presence of human plasma challenges this assumption and underlines the need to better understand how protein adsorption can either promote or prevent bacterial attachment to implant surfaces. Perhaps too much attention has been given to the bacteria–implant interaction for the initiation of implant-associated infections, while information is lacking on how aggregation of bacteria in the vicinity of implants contributes to immune evasion and biofilm formation. Previous research shows that *S. epidermidis* is less susceptible to phagocytosis when grown in human serum [31], presumably due to aggregation. Aggregates that are 10 µm in diameter are sufficiently large to escape phagocytosis [32, 33], and we show here that *S. epidermidis* forms aggregates larger than 100 µm^2^ (in 2D images) after only 2 h exposure to plasma (Fig. 2). The mechanisms responsible for aggregation may be instrumental for initiating implant-associated biofilm infections and being able to prevent – or even delay – aggregation is a potential avenue for preventing such infections.

## Supplementary Data

Supplementary material 1
