# Supplementary material for: Host factors abolish the need for polysaccharides and extracellular matrix-binding protein in Staphylococcus epidermidis biofilm formation
Source: J Med Microbiol. 2021 Jan 25;70(3):001287. doi: 10.1099/jmm.0.001287 (PMC8346721; doi:10.1099/jmm.0.001287)
Supplement: Supplementary material 1 [file jmm-70-287-s001.pdf]

Supplementary material

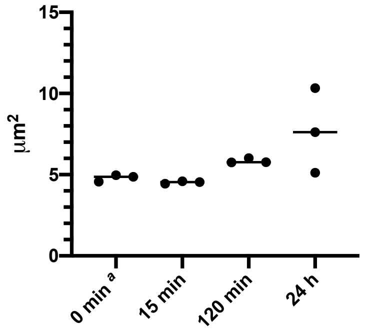

Figure S1. Aggregation of *S. epidermidis* 1585 WT in 100 % plasma
The size of aggregates formed by *S. epidermidis* 1585 WT after 24 h incubation in 100 % (v/v) human plasma (median).

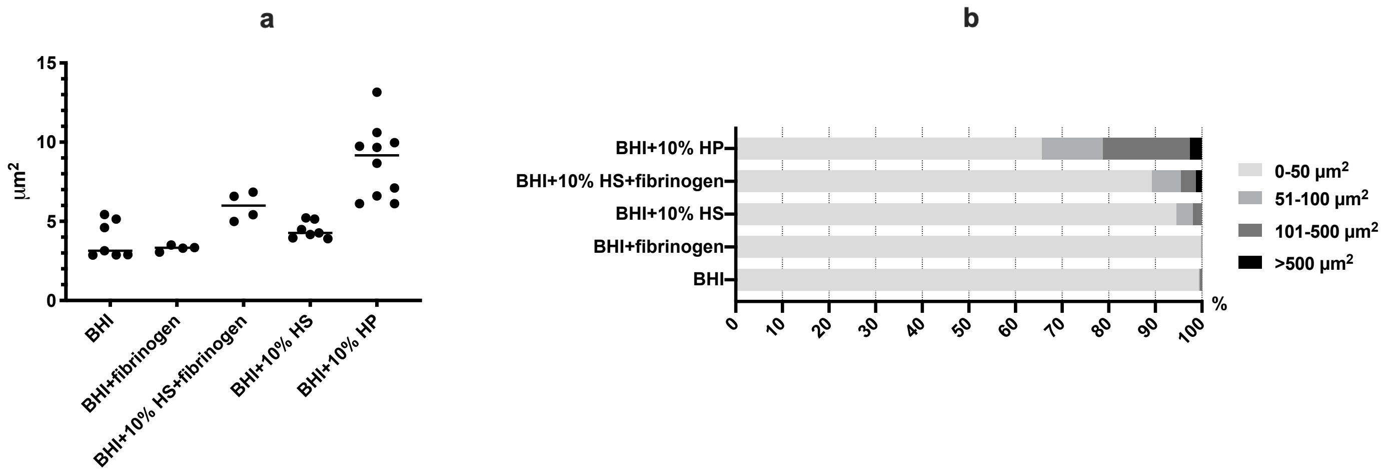

Figure S2. Effect of fibrinogen on aggregation
a) Fibrinogen alone does not induce aggregation, as aggregate size is larger for aggregates cultivated in media containing serum (HS) with fibrinogen compared with media containing BHI with fibrinogen alone (67.3 (n = 4), vs. 123.9 (n = 4), $p < 0.05$ (two-tailed) Mann-Whitney $U = 0$). Also, BHI with 10 % (v/v) serum added fibrinogen did not induce the same aggregation as BHI with plasma (HP) (123.9 (n = 4), vs. 189.5 (n = 10), $p < 0.05$ (two-tailed) Mann-Whitney $U = 5$). Each data point represents one biological replica and twenty technical replicas with > 20000 measurements. b) Aggregate size distribution at 24 h (% of biomass).

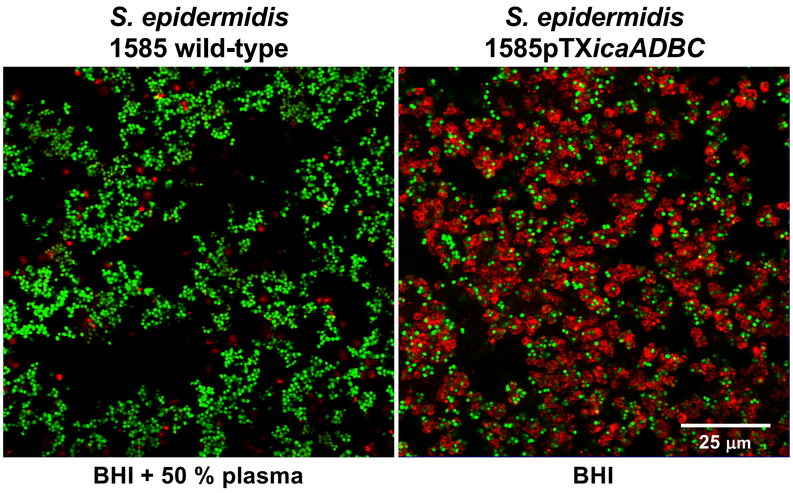

Figure S3. eDNA association with polysaccharides
2D images showing the abundance of eDNA when the biofilm matrix is Embp-dependent (left) or PIA-dependent (right). Green: live bacteria; red: extracellular DNA.
